# Molecular diversity and profile analysis of virulence-associated genes in some *Klebsiella pneumoniae* isolates

**DOI:** 10.1016/j.plabm.2020.e00152

**Published:** 2020-01-09

**Authors:** Walaa F. Alsanie

**Affiliations:** Department of Clinical Laboratory Sciences, Faculty of Applied Medical Sciences, Taif University, Saudi Arabia

**Keywords:** *Klebsiella pneumoniae*, Multidrug-resistance stains, Rep-PCR, Taif, KSA

## Abstract

The noticeable increase in the occurrence of multidrug-resistant *Klebsiella pneumoniae* strains separated from different hospitals in Taif city, (Saudi Arabia) demonstrates the limitation of antibiotics used for bacterial eradication. The aim of the present study is to detect the virulence genes in some *K. pneumoniae* isolates that collected from different hospitals in Taif governorate in Saudi Arabia. A total of 134 clinical samples were used to isolate about twenty three *K. pneumoniae* strains from various clinical specimens throw six months. They were identified by microbiological method as *K. pneumoniae* and confirmed with 16S rRNA sequencing analysis. The antimicrobial susceptibility of *K. pneumoniae* isolates was determined. The existence of virulence genes (*AcrAB, tolC, arb, OmpK35, RmpA, fimH-1, entB, K2, irP-1 and Mdtk*) were performed by PCR. The multidrug-resistant strains were detected in 16 (69.5%), that showed the presence of the most virulence genes. The multidrug-resistant isolates showed resistance against Ampicillin (96%), Amox-Clav (90%), Cephalothin (90%), Cefuroxime (90%), Ceftriaxone (85%), Aztreonam (87%), Cefepime (80%), Ceftazidime (80%), and Trim-Sulf (82%). Molecular diversity between *K. pneumoniae* isolates was determined using Rep-PCR markers technique. Thirty eight bands were resulted from the rep-PCR primers. Out of them, 31 bands were polymorphic with a polymorphism average of 81.6%. Total loci detected for each primer varied from 11 to 15 loci, and the loci size ranging from 200 to 2000 bp. These data may present novel epidemiological information regarding the clonal nature of *K. pneumoniae* separated from Taif governorate hospitals, Saudi Arabia.

## 1 Introduction

Gram-negative bacteria are potential causes of both infections acquired in hospital and community. Multiple resistance to broad spectrum antibiotics are one of the most important features. Gram-negative pathogens were successfully defeated using carbapenems, cephalosporins and fluoroquinolones in the 1980s [1]. Globally, *Klebsiella pneumoniae* is consider one of the most common nosocomial pathogens that lead to *pneumoniae*, urinary tract infections and bacteremia [2]. The increase incidence of multidrug-resistance (MDR) bacteria, such as *K. pneumoniae*, in the past few decades was due to the uncontrolled use of antimicrobial drugs as a treatment [2,3]. Gram-negative bacteria, in particular, have developed a number of mechanisms to become more resistant against antibiotics. One of these mechanisms is the horizontal gene transfer for transferring MDR genes between different types of bacteria [4]. For example, the increase incidence of *K. oxytoca, K. pneumoniae, Escherichia coli*, *Salmonella sp and Enterobacter sp* were due to the horizontal transfer of the common shared plasmids [4,5]. Another important mechanism and may be the important one for increasing the incidence of MDR bacteria is the efflux pump systems [6]. Such system in *K. pneumoniae* includes the functions of mdtK and AcrAB systems that belong to the multi antimicrobial extrusion family and resistance nodulation division of efflux pumps, respectively [6]. Also, AcrAB-TolC is another system for multidrug efflux pump which consisted of an outer-membrane channel (TolC), a secondary transporter situated in the periplasmic component (AcrA) and finally inner membrane (AcrB) [7]. This pump is important for the resistance against chloramphenicol, quinolones and tetracyclines in different MDR strains. Additionally, porins components, such as OmpK36 and OmpK35, are important for the diffusion of the antibiotics into the cells and for the sensitivity against carbapenems and cephalosporins [8].

Molecular diversity and detection of virulence genes of clinical isolates are useful assets that can reveal insight MDR K. pneumonia infections. Several studies have repeatedly used a PCR vertical component like repetitive element palindromic PCR (rep-PCR), which targets REP palindromic sequences, to compare diversity of bacterial genome [5]. In rep-PCR DNA fingerprinting, PCR amplification is achieved among heterogeneous contiguous elements to obtain DNA fingerprints that can be analyzed easily using a software program to identify patterns. Previously studies chose the rep-PCR technique because of their simplify, differentiate and cheap technique [9,10]. The rep-PCR has been used successfully to characterize *K. pneumoniae* and *E. coli* isolates [11]. Despite the fact that *K. pneumoniae* strains convey virulence related genes that may encode capsules, adhesions, lipopolysaccharides, iron acquisition systems and other harmfulness factors [12], it is not clear how these genes are linked with infection forms or antibiotic resistance.

The main objective of the present research is screening for different virulence genes like *AcrAB, tolC, arb, OmpK36, RmpA, fimH, entB, K2, irP-1 and Mdtk* in twenty three *K. pneumoniae* isolates that collected from various clinical specimens throw six months from different hospitals in Taif governorate in Saudi Arabia. Additionally, the molecular diversity between these isolates were accomplished by using Rep-PCR technique.

## 2 Materials and methods

### 2.1 Bacterial strains

Out of 134 bacterial isolates, twenty three *K. pneumoniae* isolates were obtained from inpatients of different hospital in Taif governorate, Saudi Arabia. The bacterial species were identified using the fully automated VITEK-2 COMPACT microbiology system (BioMérieux, Inc., Durham, NC, USA). The *K. pneumoniae* strain number ATCC 700603 (from ATCC, USA) was used as a positive control for the antimicrobial susceptibility test. The susceptibility test of *K. pneumoniae* against 20 types of antibiotics including amikacin, amox-Clav, ampicillin, aztreonam, cefepime, cefoxitin, ceftazidime, ceftriaxone, cefuroxime, cephalothin, ciprofloxacin, ertapenem, gentamicin, imipenem, levofloxacin, meropenem, nitrofurantoin, pipe-tazo, tigecycline, trim-sulf was determined using the recommended clinical standard (CLSI) as previously described [4].

### 2.2 The 16S rRNA gene sequencing

The genomic DNA was isolated by DNA extraction kit (Gena Bioscience, Germany) from all *K. pneumoniae* isolates according to the manufacturer’s instructions. For each isolate, one fragment of the specific gene (about 1465 bp) was amplified using primers as documented previously [4]. The specific band was punctuated by QIA quick PCR purification kit (QIAGEN, Valencia, CA, USA) and sequenced in DNA Analyzer 3146 (Applied Biosystems, USA). The sequencing results were edited and compiled using DNASTAR software (Laser gene, Madison, WI, USA). BLAST searches were performed using NCBI server (http://www.ncbi.nlm.nih.gov/blast/Blast.cgi).

### 2.3 Data analysis

The raw sequencing data of *16S* rRNA was gathered using ABI software data collection version 3.1 (ABI, Applied Biosystems). To avoid sequence mismatching, 100 bp from both ends of the *16S* rRNA gene was omitted. ClustalW aligned a uniform length of about 1365 bp in MEGA 7.0 program [13]. Genetic diversity and phylogenetic tree analysis was carried out using Maximum Likelihood method [14] applied in MEGA 7.0 program package [13].

### 2.4 Isolation of virulence genes

Ten virulence genes (*AcrAB, tolC, arb, OmpK36, RmpA, fim-H-1, entB, K2, irP-1 and Mdtk*) were checked in all strains of *K. pneumoniae*. Primers sequences (Table 1) and PCR conditions were achieved as previously reported [15]. Expected sizes of the amplicons were verified by electrophoresis in 1.5% agarose gel using 100-bp DNA ladder (MBI, Fermentas, Lithuania, USA).

**Table 1:** Primer sequences and amplicon sizes of virulence genes in *K. pneumoniae*.

Primer Name	Primer Sequence (5′→3′)	Product Size (bp)
*AcrAB-F*	ATCAGCGGCCGGATTGGTAAA	312
*AcrAB-R*	CGGGTTCGGGAAAATAGCGCG	
*tolC-F*	ATCAGCAACCCCGATCTGCGT	527
*tolC-R*	CCGGTGACTTGACGCAGTCCT	
*arb-F*	TGGGGCAAAGAGGCGCTG GAG	636
*arb-R*	CAGCCAGCGACACGGATTCTC	
*OmpK35-F*	CTCCAGCTCTAACCGTAGCG	241
*OmpK35-R*	GGTCTGTACGTAGCCGATGG	
*RmpA-F*	ACTGGGCTACCTCTGCTTCA	535
*RmpA-R*	CTTGCATGAGCCATCTTTCA	
*fimH-1F*	GCCAACGTCTACGTTAACCTG	180
*fimH-1R*	ATATTTCACGGTGCCTGAAAA	
*entB-F*	CTGCTGGGAAAAGCGATTGTC	385
*entB-R*	AAGGCGACTCAGGAGTGGCTT	
*K2-F*	GGATTATGACAGCCTCTCCT	908
*K2-R*	CGACTTGGTCCCAACAGTTT	
*irP-1-F*	TGAATCGCGGGTGTCTTATGC	238
*irP-1-R*	TCCCTCAATAAAGCCCACGCT	
*Mdtk-F*	GCGCTTAACTTCAGCTCA	543
*Mdtk-R*	GATGATAAATCCACACCAGAA	

### 2.5 Rep-PCR analysis

Three repetitive sequence primers (REP, ISSR12 and ISSR29) were used to perform the molecular diversity of *K. pneumoniae* isolates according to Wasfi et al. [15]. Rep-PCR results were obtained with the used of the following primers: REP 1R (5′-IIIICGICGICATCI GGC-3′) and 2I (5′-ICGICTTATCIGGCCTAC-3′); ISSR12 (AG)_9_T; and ISSR29 (GA)_8_A.

### 2.6 Statistical analysis

Similarity matrix and cluster analysis was achieved using an unweighted pair group method for arithmetic mean and the dendrogram was generated by NTSYS-PC program version 2.01 [16].

## 3 Results and discussion

### 3.1 Antimicrobial susceptibility

**Fig. 1:**
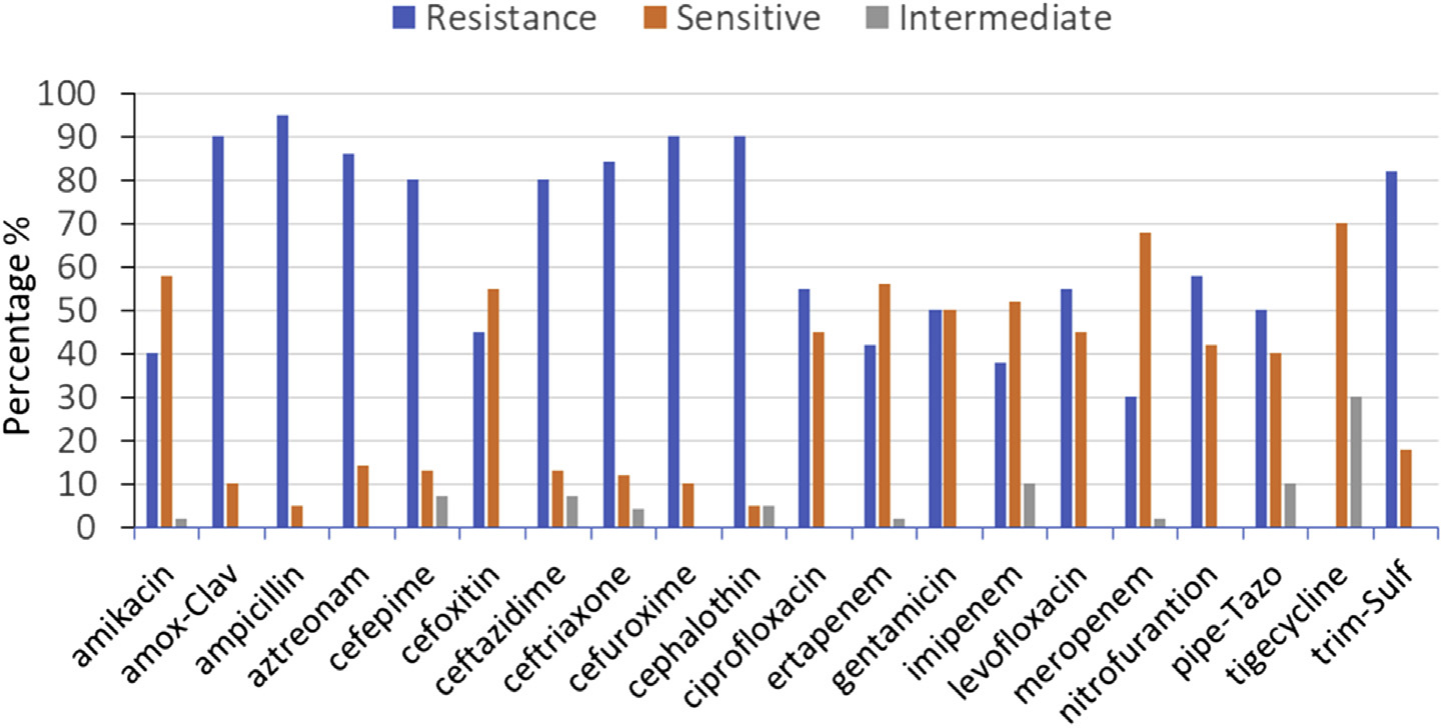
Antimicrobial resistance profiles of twenty three *K. pneumoniae* isolates against twenty antibiotics. R ​= ​resistance, S ​= ​sensitive and I ​= ​intermediate.

Twenty three *K. pneumoniae* isolates were obtained from patients of different hospitals in Taif governorate in Saudi Arabia and tested for its susceptibility against 20 types of antibiotics. The overall susceptibility, intermediate and resistance were determined and the results are showing in Fig. 1. Most *K. pneumoniae* strains showed a high percentage of resistance against Ampicillin (96%), Amox-Clav (90%), Cephalothin (90%), Cefuroxime (90%), Ceftriaxone (85%), Aztreonam (87%), Cefepime (80%), Ceftazidime (80%), and Trim-Sulf (82%). A moderate susceptible were against Tigecycline (70%), Meropenem (68%), Amikacin (57%), Imipenem (52%), Gentamicin (50%), Ertapenem (58%), and Cefoxitin (55%). On other hand, intermediate resistances were identified against Tigecycline (30%). It was reported that intermediate resistance is a compromise between resistance and sensitivity [17,18]. In this case, patients can take high doses of antibiotics. Antibiotics also play an important role in reducing the disease or death in human caused by bacterial infections. However, the uncontrolled use of antibiotics was the main cause of the emergence and spread of MDR bacterial strains [1,19].

### 3.2 Detection of virulence genes in MDR *K. pneumoniae*

**Fig. 2:**
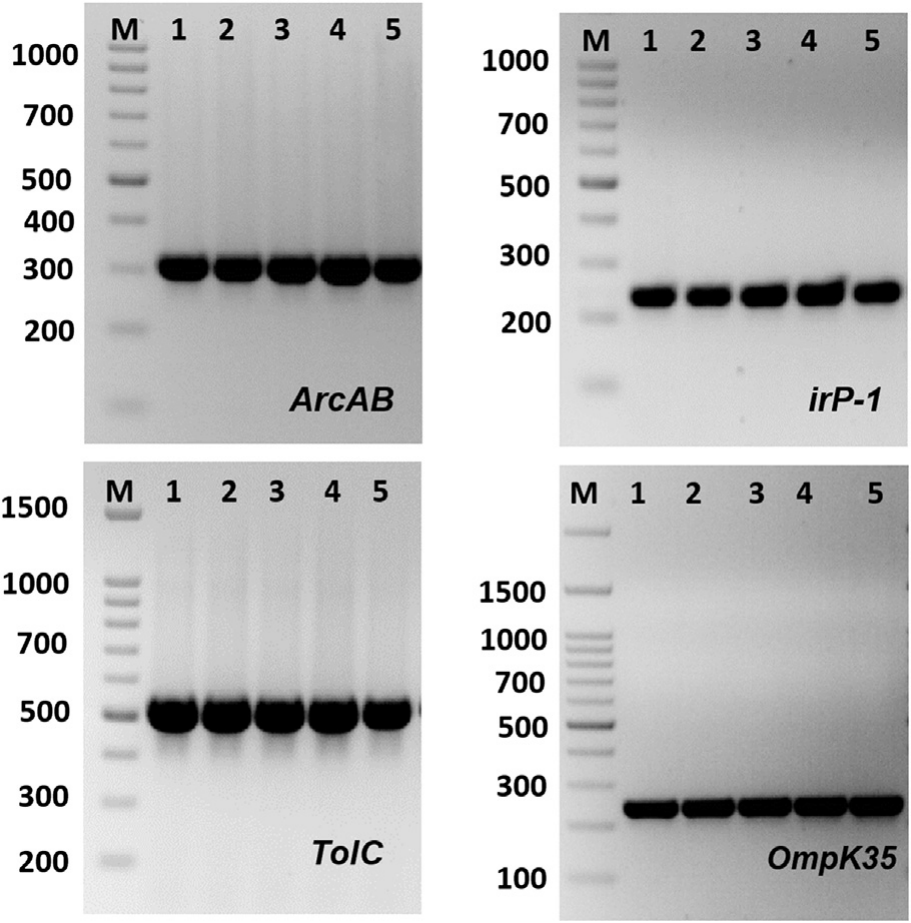
Amplification of some specific genes producing in some *K. pneumoniae* isolates by single PCR. (A) *ArcAB* gene with size about of 312 bp. (B) *irP* gene with size about of 238 bp. (C) *TolC* gene with size about of 527 bp. (D) *OmpK35* gene with size about of 241 bp. First lane on each panel is 100 bp molecular weight markers.

The existence of MDR genes are shown in Fig. 2 and Table 2. The enterobactin biosynthesis (*entB*), Yersinibactin biosynthesis (*irp-1*), *tolC, AcrAB,fumH-1,Mdtk and Ompk35* genes were exist in all of MDR *K. pneumoniae* isolates (Table 2). *K2* gene which responsible for the formation of capsule K genotypes was found in four isolates only (17.4%). Interestingly, *Klebsiella-22* and *Klebsiella-23* isolates found to have all tested virulence genes. Both *OmpK35* and *OmpK36* play a role in *K. pneumoniae* infection and virulence [8]. Deletion of *OmpK35*/*OmpK36* can result in the decrease in virulence of greatly virulent strains and can rise their susceptibility to neutrophil phagocytosis [20,21]. In the present study, *Ompk 35* porin-coding gene was identified in all the isolates *of K. pneumoniae*. A straight relationship between virulence of infectious bacteria and efflux pumps was previously described by Padilla et al. [22]. The intracellular attack of pathogenic bacteria were decreased in mutant bacterial strains that was lacking in *acrAB-tolC* efflux pumps [23]. Most strains of Enterobacteriaceae family, especially the genus of *Klebsiella*, have genes encoding iron uptake systems, including aerobactin or enterochelin systems [24]. These siderophores have dual functions as they can enhance iron uptake and inhibit T cell proliferation. The yersinibactin biosynthesis gene (*irp-1*) and enterobactin biosynthesis gene (*entB*) were identified in all MDR *K. pneumoniae* isolates. Highly pathogenic Yersinia strains have high-pathogenicity island (HPI) that include the gene *Irp-1*. This HPI is also widespread in *Klebsiella* genus especially *K. pneumonia*, *K. oxytoca* and other enterbocateria family such as and *E. coli*, Enterobacter species and Citrobacter species [3,25].

**Table 2:** Virulence genes patterns among pathogenic *K. pneumoniae* isolates.

Isolates	Present and absent of virulence genes									
	AcrAB	tolC	arb	OmpK36	RmpA	fimH	entB	K2	irP-1	Mdtk
*Klebsiella-1*	**+**	**+**	**ــ**	**+**	**+**	**+**	**+**	**+**	**+**	**+**
*Klebsiella-2*	**+**	**+**	**ــ**	**+**	**ــ**	**+**	**+**	**ــ**	**+**	**+**
*Klebsiella-3*	**+**	**+**	**ــ**	**+**	**+**	**+**	**+**	**ــ**	**+**	**+**
*Klebsiella-4*	**+**	**+**	**+**	**+**	**+**	**+**	**+**	**ــ**	**+**	**+**
*Klebsiella-5*	**+**	**+**	**+**	**+**	**ــ**	**+**	**+**	**ــ**	**+**	**+**
*Klebsiella-6*	**+**	**+**	**ــ**	**+**	**ــ**	**+**	**+**	**ــ**	**+**	**+**
*Klebsiella-7*	**+**	**+**	**ــ**	**+**	**ــ**	**+**	**+**	**ــ**	**+**	**+**
*Klebsiella-8*	**+**	**+**	**ــ**	**+**	**+**	**+**	**+**	**ــ**	**+**	**+**
*Klebsiella-9*	**+**	**+**	**ــ**	**+**	**ــ**	**+**	**+**	**ــ**	**+**	**+**
*Klebsiella-10*	**+**	**+**	**ــ**	**+**	**+**	**+**	**+**	**ــ**	**+**	**+**
*Klebsiella-11*	**+**	**+**	**ــ**	**+**	**ــ**	**+**	**+**	**ــ**	**+**	**+**
*Klebsiella-12*	**+**	**+**	**ــ**	**+**	**ــ**	**+**	**+**	**ــ**	**+**	**+**
*Klebsiella-13*	**+**	**+**	**ــ**	**+**	**+**	**+**	**+**	**+**	**+**	**ــ**
*Klebsiella-14*	**+**	**+**	**ــ**	**+**	**ــ**	**+**	**+**	**ــ**	**+**	**+**
*Klebsiella-15*	**+**	**+**	**ــ**	**+**	**ــ**	**+**	**+**	**ــ**	**+**	**+**
*Klebsiella-16*	**+**	**+**	**ــ**	**+**	**ــ**	**+**	**+**	**ــ**	**+**	**+**
*Klebsiella-17*	**+**	**+**	**ــ**	**+**	**ــ**	**+**	**+**	**ــ**	**+**	**+**
*Klebsiella-18*	**+**	**+**	**ــ**	**+**	**ــ**	**+**	**+**	**ــ**	**+**	**+**
*Klebsiella-19*	**+**	**+**	**ــ**	**+**	**ــ**	**+**	**+**	**ــ**	**+**	**+**
*Klebsiella-20*	**+**	**+**	**+**	**+**	**ــ**	**+**	**+**	**ــ**	**+**	**+**
*Klebsiella-21*	**+**	**+**	**+**	**+**	**ــ**	**+**	**+**	**ــ**	**+**	**+**
*Klebsiella-22*	**+**	**+**	**+**	**+**	**+**	**+**	**+**	**+**	**+**	**+**
*Klebsiella-23*	**+**	**+**	**+**	**+**	**+**	**+**	**+**	**+**	**+**	**+**

### 3.3 Molecular genotyping of *K. pneumoniae* isolates according to 16S-rRNA gene

**Fig. 3:**
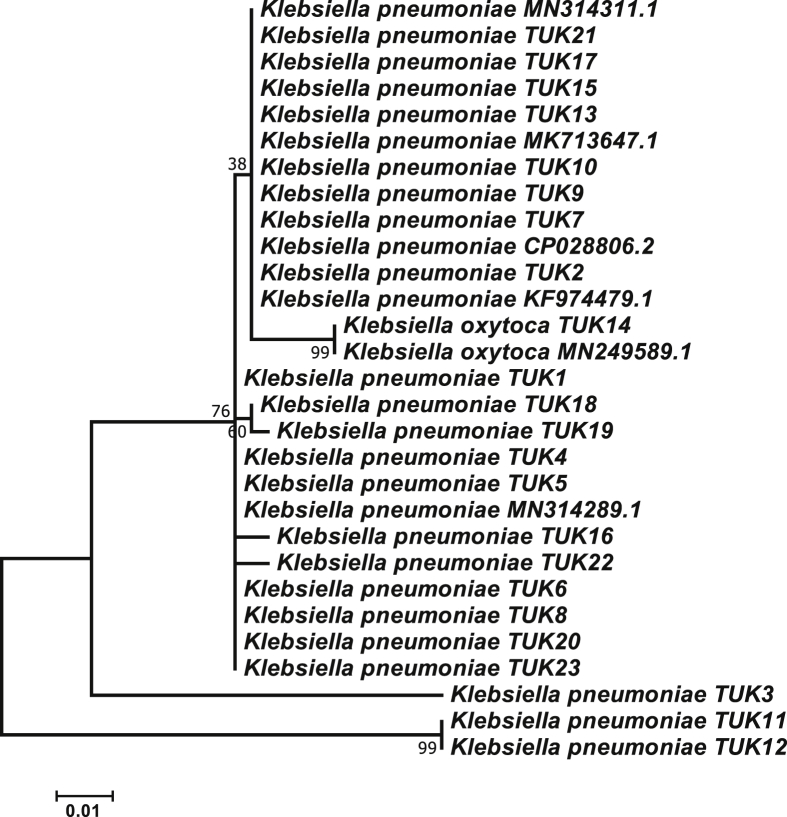
Phylogenetic tree analysis of the twenty three isolates of *K. pneumoniae* with other *K. pneumoniae* strains that blasted from NCBI based on the 1365 bp of *16s* rRNA gene sequences using the Maximum Likelihood method [14]. Numbers by nodes designate Maximum Likelihood bootstrap. The bootstrap consensus tree inferred from 1000 replicates.

We amplified and sequenced the 16S-rRNA gene of all *K. pneumoniae* isolates and then the specific fragments were aligned and compare with the available 16S-rRNA sequences for other *K. pneumoniae* isolates at the NCBI database [26]. The BLAST results showed that the partial 16S rRNA sequences are more similar to other sequences of *K. pneumoniae* strains with about 98% of similarity matrix with *K. pneumoniae* strain MN-314311 and 97% similarity matrix with *K. pneumoniae* strain MK713647 (Fig. 3). The 16S rRNA sequence contains characteristics that make it appropriate methods as a global indicator of evolution. In addition, the 16S rRNA gene sequence is a useful method for bacterial identification, where nucleotide sequences are identified in this region and compared to the available sequences of databases to obtain homogeneous matches, allowing bacterial identification of the target samples [[27], [28], [29]]. Therefore, 16S rRNA has been comprehensively used to reconstruct phylogenetic evolution of microorganisms. The genetic proximity between *K. pneumoniae* strains was also observed by various researchers [4,30] using sequencing of approximately 1200 bp of the 16S rRNA.

### 3.4 Rep-PCR and genetic distances analysis

**Fig. 4:**
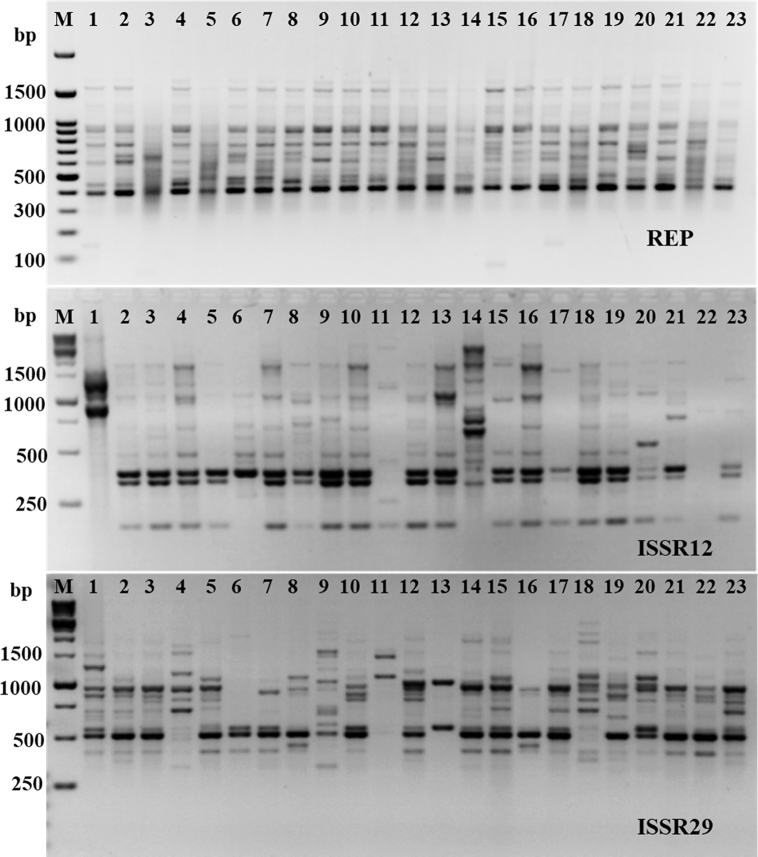
Rep-PCR profile of 23 *K. pneumoniae* isolates generated with three primers. First lane on each panel is DNA molecular weight markers.

**Fig. 5:**
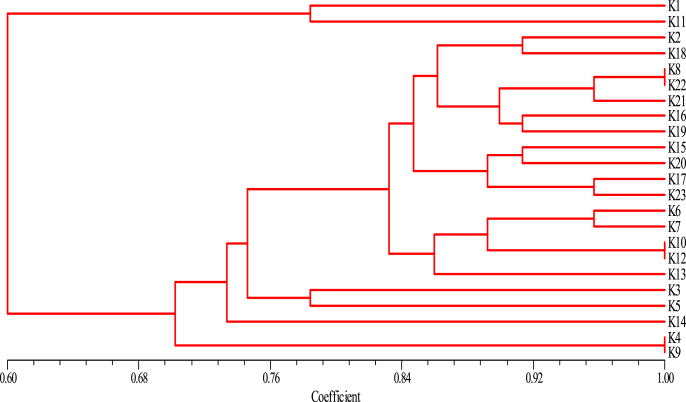
Dendrogram analysis among twenty three *K. pneumoniae* isolates (K1 to K23) generated with three rep-PCR primers.

Rep-PCR markers are effective technique for molecular characterization and correlation estimation through DNA fingerprinting [25]. The rep-PCR results were summarized in Table 3 and shown in Fig. 4. The monomorphic and polymorphic loci were produced from the PCR amplification. About 38 bands were resulted from the rep-PCR primers. Out of them, 31 bands were polymorphic bands with a polymorphism average of 81.6%. The number of total bands varied from 11 for primer ISSR12 to 15 bands for Rep-10 (Table 3). The loci sizes ranging from 200 to 2000 bp (Fig. 4). The highest polymorphism among *K. pneumoniae* isolates was produced using ISSR12 primer (100%), followed by ISSR29 primer (86.7%). On other hand, the lowest polymorphism was produced from primer REP with 54.5% (Table 3). Overall, 38 fragments produced from rep-PCR analysis were sufficient for determination of genetic similarities and constructing the phylogenetic tree for all *K. pneumoniae* isolates using UPGMA method according to Jaccard’s similarity coefficients (Fig. 5). All *K. pneumoniae* isolates were assembled into two different groups with about 40% of genetic similarity. The first group contains isolates numbers 1 and 11 only. The second group divided into two clusters, first one includes *K. pneumoniae* isolates 4 and 9, while, the second cluster includes the remaining isolates of *K. pneumoniae* (Fig. 5). The highest genetic similarity was between *K. pneumoniae* isolates 10 and 12 (Fig. 5).

**Table 3:** Polymorphic bands of each genetic primers and percentage of polymorphism in twenty three *K. pneumoniae* isolates based on the five rep-PCR primers.

Primers	Total Bands	No. of Monomorphic Bands	No. Polymorphic Bands	% Monomorphic bands	% Polymorphic bands
REP	11	5	6	45.5	54.5
ISSR12	12	0	12	0.0	100
ISSR29	15	2	13	13.3	86.7
Total	38	7	31	18.4	81.6

## 4 Conclusion

The screening of antimicrobial resistance of *K. pneumoniae* would give a better understanding for choosing the appropriate type of antimicrobial agents and avoid the existence of increasingly antimicrobial-resistant *K. pneumoniae* strains. The present data propose a temperately high predominance of antibiotic resistance in *K. pneumoniae* strains towards ampicillin, cephalothin, and cefuroxime. The present data will be very worthy to scope of antibiotics to which resistance has been gained after some time. Therefore, it better to incorporate new developing antibiotics that will be used for treatment of *K. pneumoniae* contamination.

## Funding

None.

## Declaration of competing interest

The author declare that there is no conflict of interest regarding the publication of this paper.
